# Plasma levels of the lymphangiogenic factors VEGF-C, VEGF-D, and CXCL-12 are elevated in advanced diabetic nephropathy

**DOI:** 10.1080/0886022X.2025.2536196

**Published:** 2025-08-07

**Authors:** Qingshan Tian, Fan Zhang, Zhichao Yang, Ji Miao, Hao Wu, Zhenzhong Zheng

**Affiliations:** a Department of Cardiology, The 1st Affiliated Hospital, Jiangxi Medical College, Nanchang University, Nanchang, China; b Department of Nephrology, Haidian Hospital (Haidian section of Peking University Third Hospital), Beijing, China; c Department of Cardiology, Jiu Jiang NO.1 People’s Hospital, Jiujiang, China; d Division of Endocinology, Boston Children’s Hospital, Harvard Medical School, Boston, MA, USA; e Vascular Biology Program, Boston Children’s Hospital, Department of Surgery, Harvard Medical School, Boston, MA, USA; f Cardiology, Third People’s Hospital, Shenzhen, China

**Keywords:** Type 2 diabetic kidney disease, VEGF-c, VEGF-d, CXCL-12

## Abstract

**Objective:**

Early biomarkers for diabetic nephropathy progression remain limited. This study aims to investigate whether plasma levels of VEGF-C, VEGF-D, and CXCL-12 can reflect the severity of diabetic kidney disease (DKD), and to evaluate their potential as biomarkers for disease monitoring.

**Methods:**

Patients were divided into normal albuminuria group (UmAlb < 30 mg/24 h, *n* = 30), microalbuminuria group (UmAlb 30–300 mg/24 h, *n* = 30) and macroalbuminuria group (UmAlb >300 mg/24 h, *n* = 30). Healthy individuals were included as control group (*n* = 30). Plasma levels of vascular endothelial growth factor-C (VEGF-C), vascular endothelial growth factor-D (VEGF-D), and chemokine ligand 12 (CXCL-12) were measured.

**Results:**

The plasma levels of VEGF-C, VEGF-D, and CXCL-12 were significantly increased in all type 2 diabetic kidney disease groups. Correlation analysis revealed that plasma VEGF-C, VEGF-D and CXCL-12 levels were positively correlated with plasma creatinine and urinary microalbumin. Furthermore, these levels were inversely correlated with estimated glomerular filtration rate. In order to distinguish DKD patients in the normal albuminuria group, microalbuminuria group, and macroalbuminuria group, the areas under the receiver operating characteristic curve (AUC-ROCs) of VEGF-C was 0.668 (95%CI: 0.531–0.805), 0.790 (95%CI: 0.678–0.901), and 0.850 (95%CI: 0.756–0.944), respectively. For VEGF-D, the AUC-ROCs were 0.718 (95%CI: 0.587–0.848), 0.873 (95%CI: 0.783–0.963), and 0.931 (95%CI: 0.872–0.991), respectively. Finally, for CXCL-12, the AUC-ROCs were 0.687 (95%CI: 0.554–0.820), 0.816 (95%CI: 0.710–0.921), and 0.903 (95%CI: 0.829–0.977), respectively.

**Conclusion:**

Plasma VEGF-C, VEGF-D, and CXCL-12 levels is of great value for early diagnosis and assessment of diabetic kidney disease severity. This suggests that these may serve as valuable surrogate markers for clinical outcomes in DKD.

## 1. Introduction

Diabetes mellitus (DM) is a chronic metabolic disease that seriously affects an individual’s quality of life and health [1]. The number of people with type 2 diabetes is expected to reach 439 million by 2030 [2]. Diabetes-related vascular diseases, including cardiovascular disease, cerebrovascular disease, and microvascular complications affecting the kidneys, retina, and skin, are common and the leading cause of diabetes-related complications [3]. Among these complications, diabetic kidney disease (DKD) has become an important microvascular problem, posing a substantial risk of end-stage kidney disease and requiring kidney replacement therapy [4]. DKD is characterized by microalbuminuria, progressive kidney impairment, hypertension, edema, and ultimately severe kidney failure. According to the American Kidney Data System, from 2000 to 2014, the number of patients initiating kidney replacement treatment for end-stage kidney disease (ESKD) with diabetes as the primary cause increased significantly from 41,000 cases in 2000 to 53,000 cases in 2014, with a compound annual growth rate of 1.8%. In China, a big country with diabetes, the disease burden of DKD has also increased significantly in the past decade: the latest epidemiological statistics show that the number of people with diabetes combined with chronic kidney disease (CKD) in China has reached 24.3 million, an increase of about 47% compared with 2010, among which DKD accounts for 65.2% of diabete-related CKD [5]. This increase is directly related to the increased prevalence of diabetes in both countries (13% in the US vs 11.6% in China), and also reflects the delayed onset of kidney disease due to metabolic memory effects [6].

Currently, the standard indicators for clinical diagnosis of DKD include glomerular filtration rate, urinary microalbumin excretion rate, creatinine level, urea nitrogen level and plasma cystatin level. Although these markers can effectively assess kidney function, they mainly represent late changes and do not reflect early kidney structural changes [4,7]. Therefore, there is an urgent need to identify new markers capable of early diagnosis of DKD.

With the discovery of lymphatic endothelial cell-specific markers, we have recognized the role of lymphangiogenesis in various diseases. Recent studies on mice with DKD induced by streptozotocin and high-fat diet showed that compared with the control group, the number of lymphatic vessels in the kidney cortex and medulla of the experimental group was significantly increased, suggesting that DKD may induce lymphangiogenesis, or at least induction of kidney lymphatic dilatation [8]. Vascular endothelial growth factors (VEGF) belong to the platelet-derived growth factor family and plays an important role in regulating angiogenesis and lymphangiogenesis. VEGF-C/D is the main member of the VEGF family. It binds to and activates vascular endothelial growth factor receptor 3 (VEGFR-3), promotes lymphatic endothelial cell proliferation and lymphangiogenesis, and regulates angiogenesis and lymphatic endothelial cell regeneration [9,10]. CXCL-12, also known as stromal cell-derived factor-1 (SDF-1), is a small protein called a cytokine that belongs to the chemokine protein family. CXCL-12 binds to its receptor CXCR4 to form a novel axis that regulates lymphangiogenesis. It has a strong chemotactic effect on lymphocytes and plays an important role in lymphocyte development [11,12].

This study explored that relationship between lymphangiogenesis and DKD by measuring the levels of VEGF-C, VEGF-D, and CXCL-12 in the plasma of DKD patients at different clinical stages. It further elucidates the role of lymphangiogenesis in DKD, and speculates that VEGF-C, VEGF-D, and CXCL-12 can be used as markers for the early diagnosis of DKD.

## 2. Materials and methods

### 2.1. Study population

We used a prospective observational study design and selected 90 patients with type 2 DM (T2DM) hospitalized between January 2018 and June 2019 as the study subjects. According to the Mogenson staging, patients were categorized into a normal albuminuria group (UmAlb < 30 mg/24 h, *n* = 30), microalbuminuria group (UmAlb 30–300 mg/24 h, *n* = 30), and macroalbuminuria group (UmAlb > 300 mg/24 h, *n* = 30). In addition, 30 healthy individuals were enrolled as the control group (*n* = 30).

### 2.2. Inclusion criteria

Participants were eligible if they met the following criteria: (1) aged between 18 and 90 years [13]; (2) diagnosed with T2DM according to the World Health Organization (WHO) 1999 criteria diabetes; (3) diagnostic criteria for Diabetic Kidney Disease: persistent albuminuria (≥300 mg/day) occurring in the context of diabetic retinopathy or a long duration of diabetes., reduced estimated glomerular filtration rate (eGFR <60 mL/min/1.73 m^2^) [14].

### 2.3. Exclusion criteria

The exclusion criteria included: (1) recent urinary tract infection or kidney injury and kidney transplantation resulting from cardiovascular disease, liver disease and autoimmune disorders; (2) diabetic ketoacidosis, hyperosmolar hyperglycemia syndrome, and acute infections requiring treatment; (3) DKD combined with tumor disease; (4) pregnancy and/or lactation; (5) DKD combined with acute myocardial infarction or cardiac insufficiency; and (6) DKD and complicated with autoimmune diseases and inflammation in other parts.

### 2.4. Basic data collection

Questionnaires were developed to collect patient baseline information, including gender, age, blood pressure (SBP and DBP), fasting blood glucose (FBG) level, triglyceride (TG) level, total cholesterol (TC) level, low density lipoprotein cholesterol (LDL-C) level, high-density lipoprotein cholesterol (HDL-C) level, creatinine (Cr) level, glycosylated hemoglobin (HbA1c) level, urine microalbumin (UmAlb) level, estimated glomerular filtration rate (eGFR), etc. All indicators were based on the scores of the first examination after admission.

### 2.5. Measurement of plasma VEGF-C, VEGF-D, and CXCL-12 levels

On the second day of admission, 5 mL of venous blood was collected using a vacuum anticoagulant tube while the patient was fasting. Blood samples were centrifuged at 3000 rpm for 10 min. The upper plasma fractions were then separated into eppendorf tubes and stored in a −80 °C freezer.

### 2.6. Measurement of serum VEGF-C, VEGF-D, and CXCL12 levels

The experimental method utilized enzyme-linked immunosorbent assay (ELISA) for detection, and all reagents and materials used in the experiment were purchased from Nanjing Pars Biotechnology Co., Ltd.

### 2.7. Statistical analysis

All data were analyzed using SPSS 17.0 software, and graphs were created using GraphPad prism 9.0 software. First, the measured data were tested for normality. Normally distributed measurement data are expressed as mean ± standard deviation. Analysis of variance using Levene’s method with homogeneity of variance test was used for comparisons between groups. The LSD-t test was used for comparison between groups, and the Tamhane T2 test was applied for unequal variance. Count data were analyzed using the chi-square test. Pearson correlation analysis was used to evaluate the correlation between observed indicators. Receiver operating curves (ROCs) were drawn to analyze the value of plasma levels of VEGF-C, VEGF-D, and CXCL-12 in evaluating the severity ofDKD. A significane level of *p* < 0.05 was considered statistically significant.

## 3. Result

### 3.1. Basic data comparison

A total of 90 DKD patients were enrolled in this study, including 60 males and 30 females, aged 37–87 years old, with an average age of 60.22 ± 11.04 years. Each study group included 30 patients. There were 25 males and 5 females in the healthy control group, aged from 23 to 84 years old, with an average age of 60.97 ± 16.52 years. There were no statistically significant differences in sex and age between the groups (*p* > 0.05), indicating that the basic data of each group were balanced and comparable (Table 1).

**Table 1.** Basic data of DKD patients in different stages and comparison with healthy controls.

Group	No. of patients	Sex		Age (years)	
	(*N* = 120)	Male (*N* = 85)	Female (*N* = 35)	Range	$\bar{x}+s$
Normal albuminuria group	30	17	13	41–83	59.56 ± 11.82
Microalbuminuria group	30	23	7	37–75	59.37 ± 9.29
Macroalbuminuria group	30	20	10	37–87	61.73 ± 12.01
Control group	30	25	5	23–84	60.97 ± 16.52
*c*^2^/*F*-value		5.929			0.24
*p*-Value		0.115			0.868

### 3.2. Comparison of plasma VEGF-C, VEGF-D, and CXCL-12 levels in different groups DKD patient groups

One-way ANOVA analysis of variance showed that there were statistically significant differences in the relative expression levels of plasma VEGF-C, VEGF-D, and CXCL-12 between the macroalbuminuria group, the microalbuminuria group, the normal proteinuria group, and the control group (*p* < 0.01). Pairwise comparisons were performed using LSD-t test.

Compared with the control group, the expression levels of plasma VEGF-C, VEGF-D, and CXCL-12 in the macroalbuminuria group, microalbuminuria group, and normoalbuminuria group gradually increased with the progression of DKD, in the following order: macroalbuminuria group > microalbuminuria group > normal albuminuria group > control group. The difference was significant (*p* < 0.05), as shown in Table 2.

**Table 2.** Comparison of plasma levels of VEGF-C, VEGF-D and C XCL-12 in different stages of DKD.

Group	No. of patients	VEGF-C	VEGF-D	CXCL-12
	(*N* = 120)	pg/mL	pg/mL	pg/mL
Normal albuminuria group	30	239.54 ± 58.18	265.18 ± 65.22	625.10 ± 172.17
Microalbuminuria group	30	280.00 ± 62.78	321.02 ± 68.64	727.87 ± 169.97
Macroalbuminuria group	30	303.46 ± 62.34	359.44 ± 72.86	823.82 ± 172.46
Control group	30	201.75 ± 66.10	212.62 ± 64.61	510.70 ± 154.89
*F*-value		15.51	26.858	19.38
*p*-Value		<0.001	<0.001	<0.001

### 3.3. The relationship between plasma VEGF-C, VEGF-D, and CXCL-12 levels and major clinical indicators of DKD

**Figure 1.**
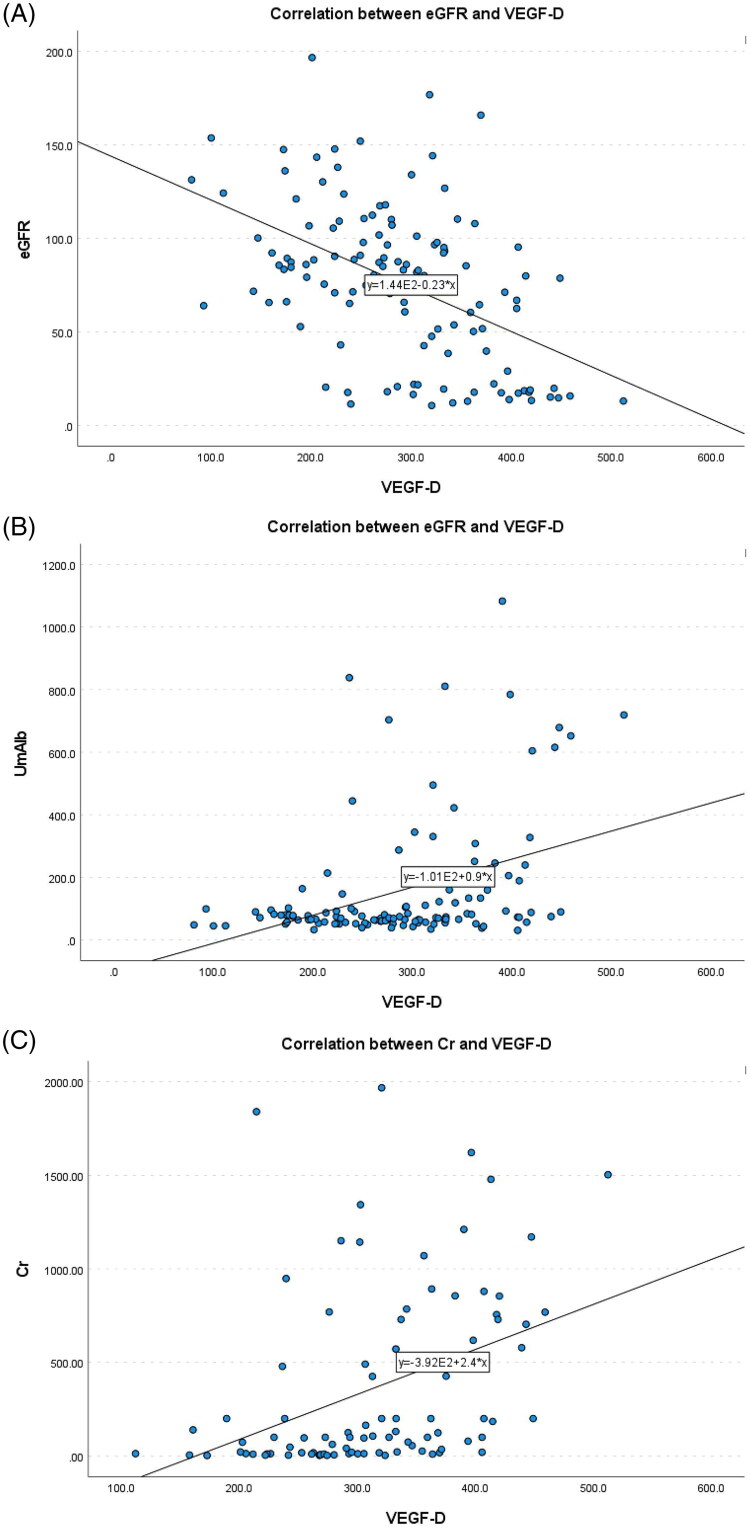
(A) The correlation between eGFR and VEGF-C. (B) The correlation between Cr and VEGF-C. (C) The correlation between UmAlb and VEGF-C.

**Figure 2.**
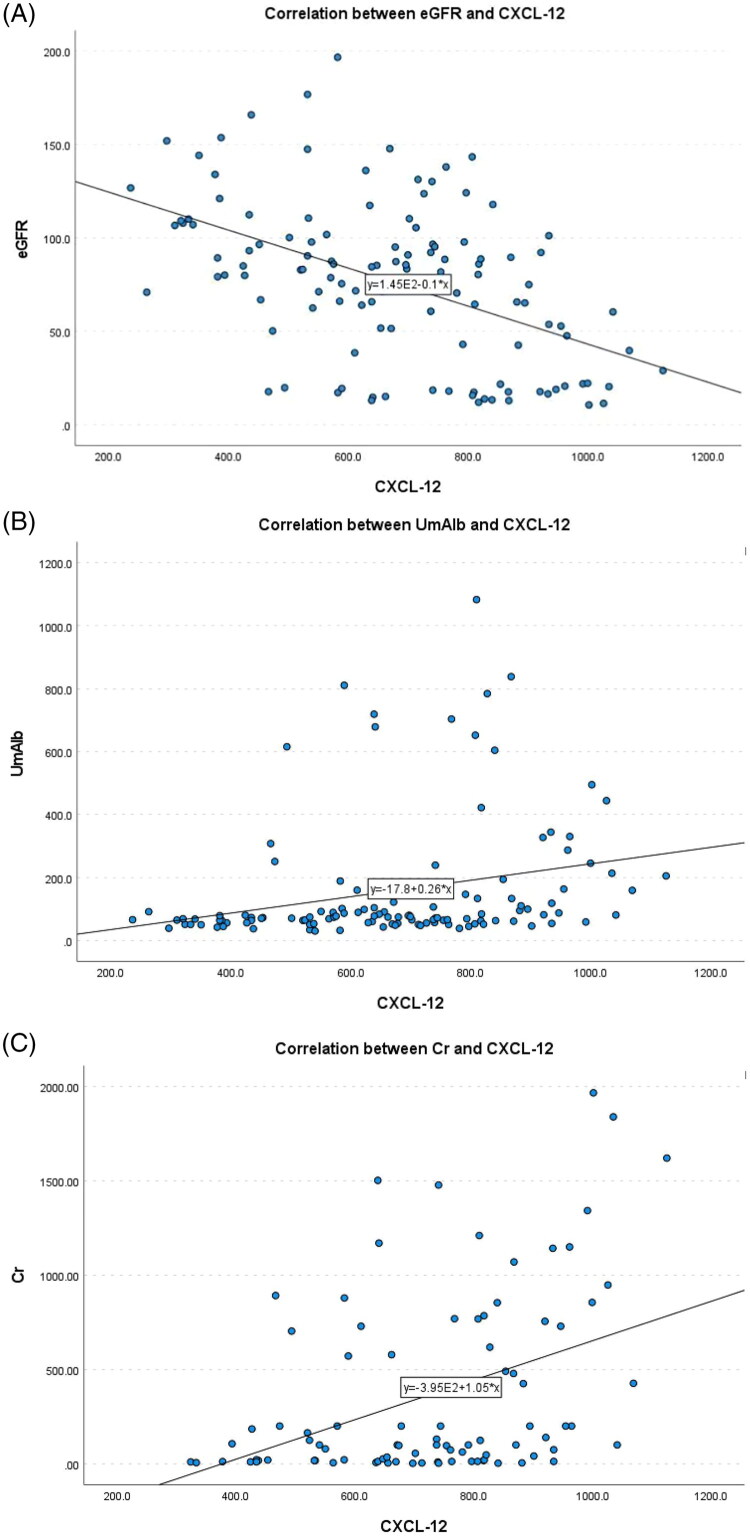
(A) The correlation between eGFR and VEGF-D. (B) the correlation between Cr and VEGF-D. (C) the correlation between UmAlb and VEGF-D.

**Figure 3.**
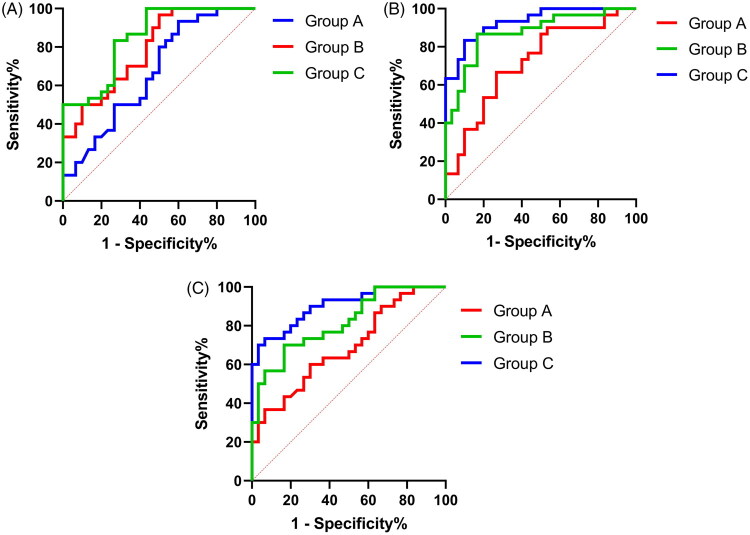
(A) The correlation between eGFR and CXCL-12. (B) the correlation between Cr and CXCL-12. (C) the correlation between UmAlb and CXCL-12.

Correlation analysis revealed that there was no significant correlation between plasma VEGF-C, VEGF-D, and CXCL-12 levels in DKD patients and age, gender, systolic blood pressure, diastolic blood pressure, fasting blood glucose, triglycerides, and total cholesterol levels. *p* > 0.05). Specifically, plasma *VEGF-C* levels were significantly negatively correlated with *eGFR* (−0.366, *p* < 0.01), and significantly positively correlated with *UmAlb and Cr* levels (0.357, 0.283, respectively, *p* < 0.01), as shown in Figure 1(A–C). Plasma *VEGF-D* levels were significantly negativel correlated with *eGFR* (−0.478, *p* < 0.01), and significantly positively correlated with *UmAlb and Cr* levels (0.479, 0.43, respectively, *p* < 0.01), as shown in Figure 2(A–C). Plasma *CXCL-12* levels were significantly negatively correlated with *eGFR* (−0.486, *p* < 0.01), and significantly positively correlated with *UmAlb and Cr* levels (0.396, 0.431, *p* < 0.01, respectively), as shown in Figure 3(A–C) (Table 3).

**Table 3.** Relationship between plasma VEGF-C, VEGF-D and CXCL-12levels and clinical indicators of DKD.

Clinical parameters	VEGF-C		VEGF-D		CXCL-12	
	*r*	*p*	*r*	*p*	*r*	*p*
Age (years)	−0.062	>0.05	0.016	>0.05	0.122	>0.05
Sex (male/female)	0.057	>0.05	0.140	>0.05*	−0.176	>0.05
SBP (mmHg)	0.061	>0.05	0.144	>0.05	0.294	<0.05
DBP (mmHg)	−0.085	>0.05	0.103	>0.05	0.056	>0.05
Cr (μmol/L)	0.283	<0.01	0.436	<0.01	0.413	<0.01
eGFR (mL/min/1.73 m^2^)	−0.366	<0.01	−0.478	<0.01	−0.486	<0.01
FBS (mmol/L)	0.139	>0.05	0.225	>0.05	0.141	>0.05
TG (mmol/L)	0.115	>0.05	0.140	>0.05	−0.032	>0.05
TC (mmol/L)	−0.107	>0.05	−0.013	>0.05	0.140	>0.05
LDL-C (mmol/L)	−0.162	>0.05	−0.068	>0.05	0.161	>0.05
UmAlb (mg/24h)	0.194	>0.05	0.367	<0.01*	0.401	<0.01

*Note*. SBP: systolic blood pressure; DBP: diastolic blood pressure; Cr: creatinine; eGFR: estimated glomerular filtration rate; FBS: fasting blood sugar; TG: triglyceride; TC: total cholesterol; LDL-C: low density lipoprotein cholesterol; UmAlb: urinary microalbumin.

### 3.4. The diagnostic value of plasma VEGF-C, VEGF-D, and CXCL-12 levels in DKD

**Figure 4.**
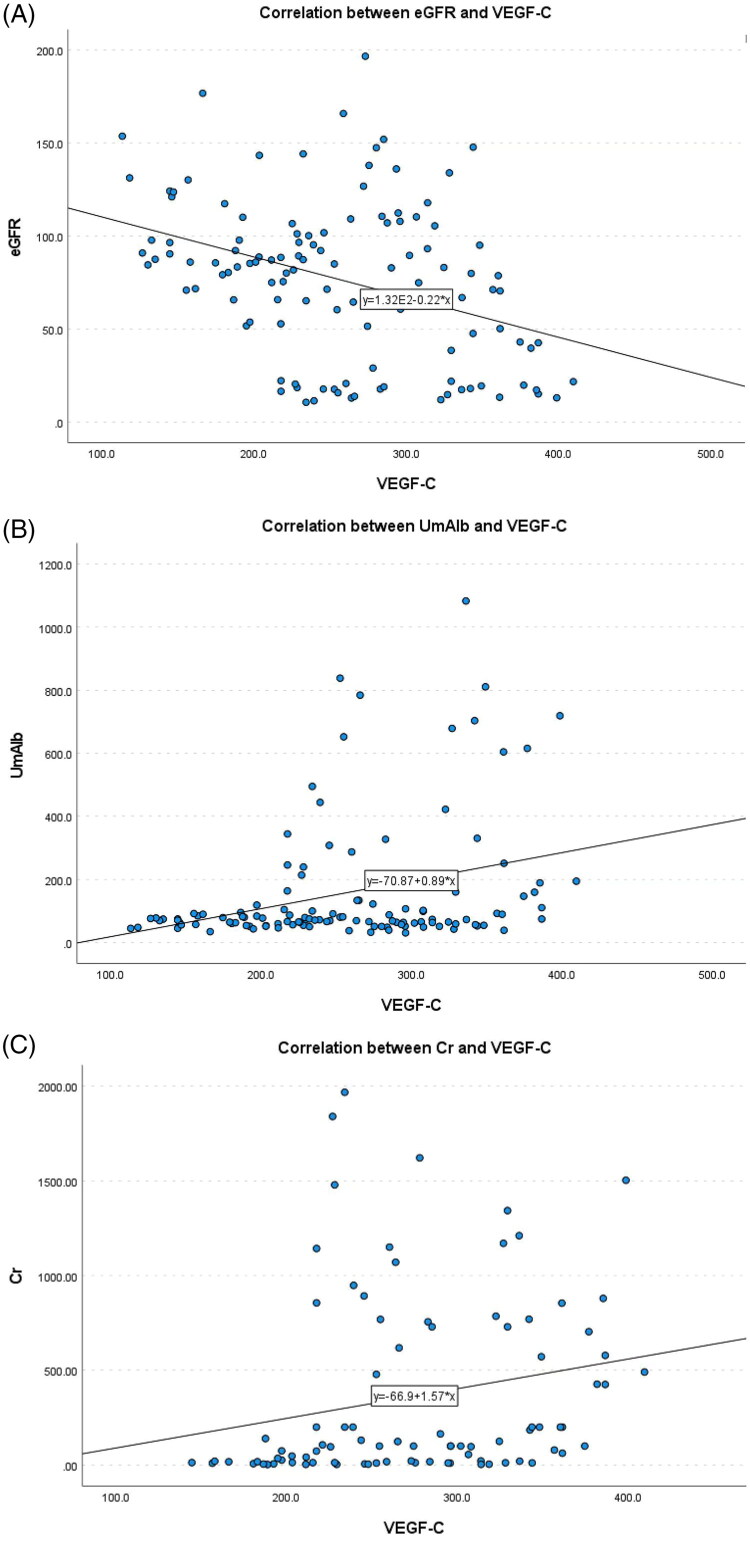
(A) The ROC curves of plasma VEGF-C in different groups for diagnosis of DKD. (B) The ROC curves of plasma VEGF-D in different groups for diagnosis of DKD. (C) The ROC curves of plasma CXCL-12 in different groups for diagnosis of DKD.

ROC curve analysis demonstrated that the areas under the ROC curves (AUCs) of the normal albuminuria group, microalbuminuria group, and macroalbuminuria group were 0.668 (95%CI: 0.531–0.805), 0.790 (95%CI: 0.678–0.901), and 0.850 (95%CI: 0.756–0.944); when the cutoff values were 154.5, 190.0 and 212.60 pg/mL. The sensitivity values were 93.3%, 96.7%, and 100.0%, respectively, and the specificities were 40.0%, 50.0%, and 56.7%, respectively. Notably, the plasma levels of VEGF-D and CXCL-12 levels used to assess the ROC curve area in the macroalbuminuria group, microalbuminuria group, and normal albuminuria group were higher than the VEGF-C level This suggests that plasma levels of VEGF-C, VEGF-D, and CXCL-12 have some value for early diagnosis of DKD and assessment of disease severity, as shown in Figure 4(A–C), and detailed in Tables 4–6.

**Table 4.** Value of plasma levels of VEGF-C in assessing severity of DKD.

VEGF- C	AUC	*SE*	*p*-Value	95% CI	Cutoff value	Sensitivity (%)	Specificity (%)
Group A	0.668	0.070	0.025	0.531–0.805	154.500	93.300	40.000
Group B	0.790	0.057	<0.01	0.679–0.902	190.000	96.700	50.000
Group C	0.850	0.048	<0.01	0.756–0.944	212.600	100.000	56.700

**Table 5.** Value of plasma levels of VEGF-D in assessing severity of DKD.

VEGF-D	AUC	SE	*p*-Value	95% CI	Cutoff value	Sensitivity (%)	Specificity (%)
Group A	0.718	0.067	0.004	0.587–0.848	244.000	66.700	73.300
Group B	0.873	0.046	<0.010	0.783–0.963	274.000	86.700	83.300
Group C	0.931	0.030	<0.010	0.872–0.991	293.000	83.300	90.000

**Table 6.** Value of plasma levels of CXCL-12 in assessing severity of DKD.

CXCL- 12	AUC	SE	*p*-Value	95% CI	Cutoff value	Sensitivity (%)	Specificity (%)
Group A	0.687	0.068	0.013	0.554–0.820	589.000	60.000	70.000
Group B	0.816	0.054	<0.01	0.710–0.921	661.00	70.000	83.300
Group C	0.903	0.038	<0.01	0.829–0.977	741.00	70.000	96.700

**Table 7.** Comparison of Cr and eGFR among DKD patients at different stages and healthy controls.

Group	No. of patients (*N* = 120)	Cr (µmol/L)	eGFR (mL/min/1.73 m^2^)
Normal albuminuria group	30	62.28 ± 18.16	110.29 ± 32.98
Microalbuminuria group	30	92.03 ± 63.88	110.29 ± 32.98^ab^
Macroalbuminuria group	30	406.14 ± 277.85^abc^	19.60 ± 8.01^abc^
Control group	30	69.30 ± 15.45	102.69 ± 26.37
*F* value		40.54	94.68
*p* Value		<0.001	<0.001

*Note*. ^a^Compared with the control group, *p* < 0.05. ^b^Compared with the Normal albuminuria group, *p* < 0.05. ^c^Compared with the Micro albuminuria group, *p* < 0.05.

## 4. Discussion

The lymphatic system is an important auxiliary system for tissue fluid reflux, maintainins dynamic fluid balance, aids lipid absorption, and promotes immune monitoring. In various pathological conditions, such as inflammatory reactions, tissue wound repair, organ transplantation, and tumors, lymphatic vessel formation can be reactivated [15,16]. Currently, research on lymphangiogenesis is predominantly focused on cancer, and the mechanism of lymphangiogenesis in diabetic kidney disease (DKD) remains unclear. Inflammation is a known contributor to lymphangiogenesis, with inflammatory factors, such as interleukin-1 and tumor necrosis factor alpha, as well as macrophages, increasing the production of vascular endothelial factors and promoting lymphangiogenesis. Although research on lymphangiogenesis has mainly focused on cancer, its mechanism in DKD remains unclear. Inflammation is considered a key factor in lymphangiogenesis, in which inflammatory factors and macrophages contribute to the production of endothelial factors and promote lymphangiogenesis [17,18]. Recent studies in human kidney disease have shown that lymphangiogenesis is affected by the duration of inflammation and progression of fibrosis, rather than acute inflammation, particularly in the setting of chronic interstitial inflammation in DKD. DKD, characterized by chronic interstitial inflammation, exhibits mesangial dilatation, podocyte loss, glomerular proliferation, glomerular basement membrane thickening, and tubular epithelial cell dysfunction, leading to glomerular capillary occlusion. As a response to kidney edema and hypertension compensation, lymphatic vessel formation becomes crucial [8,19,20].

DKD, a severe complication of diabetes that initially manifests as microalbuminuria. Persistent metabolic and hemodynamic perturbations associated with diabetes lead to inflammatory changes in the kidney that promote the transition from injury to repair and ultimately result in kidney fibrosis, particularly in stages III and IV [21,22]. Numerous studies have demonstrated an association between kidney fibrosis and lymphangiogenesis. For example, induction of kidney fibrosis in mice by unilateral ureteral obstruction showed a positive correlation between kidney lymphangiogenesis and fibrosis [23]. To explore the relationship between lymphangiogenesis and kidney fibrosis, another study analyzed inflammation, fibrosis, lymphangiogenesis, and growth factor expression in rats with unilateral ureteral obstruction. Levels of transforming growth factor-β1 (TGF-β1) and VEGF-C were found to be elevated in kidney tubular epithelial cells and monocytes and peaked 14 days after ureteral obstruction [24]. Pathological examination of kidney biopsy specimens also supports the importance of lymphangiogenesis in DKD. One study found that patients with tubulointerstitial disease had an increased number of lymphatic vessels that correlated more strongly with areas of fibrosis than with areas of inflammation. Lymphangiogenesis is significantly higher in patients with DKD than in other kidney diseases [25]. Therefore, lymphangiogenesis is a common feature of tubulointerstitial fibrosis. In addition, in DKD, cytokines activate VEGF-C/VEGFR-3, a primary lymphangiogenic pathway [26]. However, VEGF-C exhibits only ∼50% specificity in DKD due to shared upregulation in cancers and other kidney diseases, limiting its diagnostic value. Complementary factors like VEGF-D and IL-6/IL-17 may amplify or compensate for VEGF-C, indicating a complex regulatory network. In addition, VEGF-C (∼50%) specificity in DKD precludes its standalone use. Integrating it with urinary TGF-β1 or lymphatic imaging could improve diagnostic precision.

Some studies have shown that the drugs required for treatment also affect lymphangiogenesis to some extent. ACEIs/ARBs not only reduce blood pressure but also attenuate kidney fibrosis by inhibiting the TGF-β/Smad pathway. Animal studies demonstrate that ramipril treatment downregulates kidney VEGF-C expression by 34% in DKD rats (*p* < 0.05) [27], indicating potential indirect regulation of lymphangiogenic activity. Empagliflozin may suppress HIF-1α signaling by ameliorating tubular hypoxia, thereby reducing VEGF-D synthesis and release [28]. The lack of stratified analysis for these novel antidiabetic agents in our study may introduce confounding effects on biomarker levels. Hyperinsulinemia could promote VEGFR-3 phosphorylation *via* PI3K/Akt pathway activation, enhancing lymphatic endothelial cell migration [29]. Although 62% of our cohort received insulin therapy, quantitative analysis of its impact on inflammatory markers like CXCL-12 was not performed.

The advantages and disadvantages of lymphangiogenesis in DKD remain controversial. Lymphatic vessels are important regulators of fluid balance, immune cell transport, and immune recognition and play a key role in various diseases. DKD is a major cause of end-stage kidney disease worldwide, and oxidative stress and inflammation caused by hyperglycemia play a crucial role in its occurrence and progression. Lymphangiogenesis is an important component of the inflammatory process in tissues and organs. Numerous studies have shown that an expanded lymphatic system is necessary to resolve inflammation, with lymphatic vessels playing a crucial role in fluid clearance and immune cell transport, ultimately reducing or alleviating inflammation [30,31]. However, recent studies suggest that lymphangiogenesis may not always be beneficial to the progression of DKD. While functional lymphatics are critical for maintaining fluid balance and immune surveillance, deranged lymphangiectasia leads to failure of immune cell clearance, leading to chronic inflammation. Selective inhibition of VEGFR-3 to suppress lymphoproliferation in DKD mice has been shown to reduce plasma cholesterol levels, free fatty acids, and proteinuria, thereby reducing kidney inflammation and oxidative stress and attenuating kidney fibrosis [32]. Another study questioned the involvement of lymphatic function in DKD. As chronic inflammation in DKD develops, the expression of various inflammatory factors becomes unbalanced, leading to excessive lymphatic vessel growth, structural integrity, and dysfunction. Research shows that reducing kidney lipotoxicity can alleviate kidney lymphatic dysfunction in DKD, thereby reducing kidney inflammation and fibrosis [33].

## 5. Conclusion

The results of this study indicate a strong association between plasma levels of VEGF-C, VEGF-D, and CXCL-12 and DKD. As DKD progresses, the expression levels of plasma VEGF-C [34], VEGF-D [35], and CXCL-12 [36] gradually increase. These findings are consistent with a large body of research supporting the notion that lymphangiogenesis accompanies the development of DKD and is positively correlated with disease severity. This finding may reflect the active pathological processes of diabetic nephropathy; however, the possibility that these markers accumulate due to reduced renal clearance as kidney function declines cannot be ruled out (Table 7). ROC curve analysis demonstrated that plasma VEGF-C, VEGF-D, and CXCL-12 levels have diagnostic value in the early stages of DKD and can at least be used to predict disease progression. Although the debate on whether lymphangiogenesis is beneficial or harmful in DKD continues, some studies have shown that inhibiting lymphangiogenesis can partially alleviate inflammation and fibrosis in DKD, providing new insights into treatment. However, it is important to note that further research and clinical trials are needed to confirm these findings.

In addition, the limitations of this study include: 1) Cross-sectional nature of data; 2) Single-day blood and urine sampling; 3) Inpatient setting of DM patients; 4) Potential influence of unmeasured confounding factors; 5) the absence of blood urea nitrogen (BUN) measurement.Although studies have focused on specific biomarkers associated with lymphangiogenesis (vascular endothelial growth factor-C, vascular endothelial growth factor-D, and CXCL-12), blood urea nitrogen is an important parameter for assessing kidney function. Since kidney function may influence the mechanisms being studied in relation to lymphangiogenesis, the absence of this metric may limit a more complete understanding of the overall physiologic context. Therefore, in the future, we will also continue to include BUN based on this study and conduct a multi-country, multi-sample, multi-center study.

## Data Availability

For data, please contact the first author: Qingshan Tian; Email: ndyfy10093@ncu.edu.cn.
